# Severe and rapidly progressing cognitive phenotype in a SCA17-family with only marginally expanded CAG/CAA repeats in the TATA-box binding protein gene: A case report

**DOI:** 10.1186/1471-2377-12-73

**Published:** 2012-08-13

**Authors:** Troels Tolstrup Nielsen, Skirmante Mardosiene, Annemette Løkkegaard, Jette Stokholm, Susanne Ehrenfels, Sara Bech, Lars Friberg, Jens Kellberg Nielsen, Jørgen E Nielsen

**Affiliations:** 1 Memory Disorders Research Group, Neurogenetics Clinic, Department of Neurology, Rigshospitalet, Copenhagen University Hospital, Copenhagen, Denmark; 2 Department of Cellular and Molecular Medicine, Section of Neurogenetics, The Panum Institute, University of Copenhagen, Copenhagen, Denmark; 3 Department of Neurology, Bispebjerg Hospital, Copenhagen University Hospital, Copenhagen, Denmark; 4 Department of Clinical Physiology and Nuclear Medicine, Bispebjerg Hospital, Copenhagen University Hospital, Copenhagen, Denmark; 5 Department of Radiology, Bispebjerg Hospital, Copenhagen University Hospital, Copenhagen, Denmark

**Keywords:** Spinocerebellar ataxia type 17, Dementia, Short CAG repeat expansion

## Abstract

**Background:**

The autosomal dominant spinocerebellar ataxias (SCAs) confine a group of rare and heterogeneous disorders, which present with progressive ataxia and numerous other features e.g. peripheral neuropathy, macular degeneration and cognitive impairment, and a subset of these disorders is caused by CAG-repeat expansions in their respective genes. The diagnosing of the SCAs is often difficult due to the phenotypic overlap among several of the subtypes and with other neurodegenerative disorders e.g. Huntington’s disease.

**Case presentation:**

We report a family in which the proband had rapidly progressing cognitive decline and only subtle cerebellar symptoms from age 42. Sequencing of the TATA-box binding protein gene revealed a modest elongation of the CAG/CAA-repeat of only two repeats above the non-pathogenic threshold of 41, confirming a diagnosis of SCA17. Normally, repeats within this range show reduced penetrance and result in a milder disease course with slower progression and later age of onset. Thus, this case presented with an unusual phenotype.

**Conclusions:**

The current case highlights the diagnostic challenge of neurodegenerative disorders and the need for a thorough clinical and paraclinical examination of patients presenting with rapid cognitive decline to make a precise diagnosis on which further genetic counseling and initiation of treatment modalities can be based.

## Background

Autosomal dominant spinocerebellar ataxias (SCAs) confine a group of rare and heterogeneous hereditary disorders which present with progressive ataxia and numerous other features e.g. peripheral neuropathy, macular degeneration and cognitive impairment [1]. 32 subtypes of the autosomal dominantly inherited ataxias are known, and 8 of these (SCA1, 2, 3, 6, 7, 12, 17 and Dentato-rubro-pallido-Luysian atrophy (DRPLA)) are caused by CAG repeat expansions in the respective genes underlying disease [1,2]. The expanded CAG repeat results in an elongated polyglutamine tract that interferes with normal protein function [1,3]. SCA17, a member of the group of polyglutaminopathies, is caused by a CAG/CAA repeat expansion in the gene encoding the TATA-box binding protein (*TBP*), which is a general transcription factor crucial for normal cellular function and development [4]. The normal range of glutamine stretches in TBP is 25–41. Stretches of 49 or more glutamines are disease causing with full penetrance, whereas stretches of 42–48 glutamines are associated with reduced penetrance [5]. The clinical diagnosing of SCA17 patients is complex. Typically ataxic gait and dysarthria are the most prominent signs, but other features include personality changes, depression, cognitive impairment and basal ganglia dysfunction. Hence, SCA17 is also classified as a Huntington's disease like syndrome. All symptoms can be present at different times and to a varying degree during the course of disease, and because there is a significant phenotypic overlap with other neurodegenerative disorders such as Huntington’s disease (HD) as well as with other SCAs, diagnosis based on the clinical presentation remains a considerable challenge [6]. The age of onset in SCA17 is highly variable (between 3 and 55 years), and although there is some correlation between age of onset and the number of CAG/CAA repeats it seems to be less pronounced compared to other polyglutamine disorders e.g. HD. Similarly, anticipation is infrequently reported in SCA17 families due to an interruption of the CAG repeat sequence by a CAACAGCAA sequence that seems to stabilize the repeat during replication [7,8]. A milder phenotype with later age of onset and slower disease progression seems to be associated with repeat lengths in the shorter end of the reduced penetrance range (42–44 CAG/CAA repeats) while a more severe and rapidly progressing phenotype is seen in patients with longer repeats [4,8-10]. Here we report on a family in which the proband shows only very modest cerebellar symptoms consisting of mild ataxia and dysarthria. Interestingly, he has severe cognitive impairment with rapid progression although the CAG/CAA repeat in the TBP gene is expanded by only two repeats above the non-pathogenic threshold of 41 repeats.

## Case presentation

### Family history

**Figure 1:**
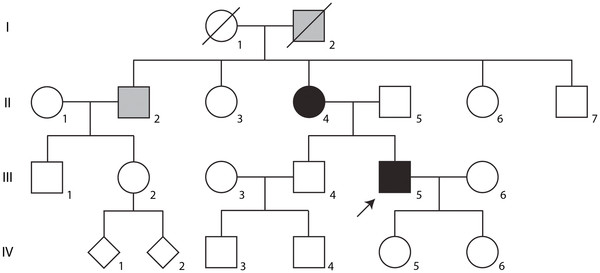
**Pedigree of the SCA17 family.** Individuals marked with solid black have a molecular diagnosis of SCA17 accompanied by clinical manifestations of neurological disorders. Individuals marked with gray shading have a clinical diagnosis of a neurological disorder, which has not been confirmed on the molecular level. The proband is marked by an arrow.

**Figure 2:**
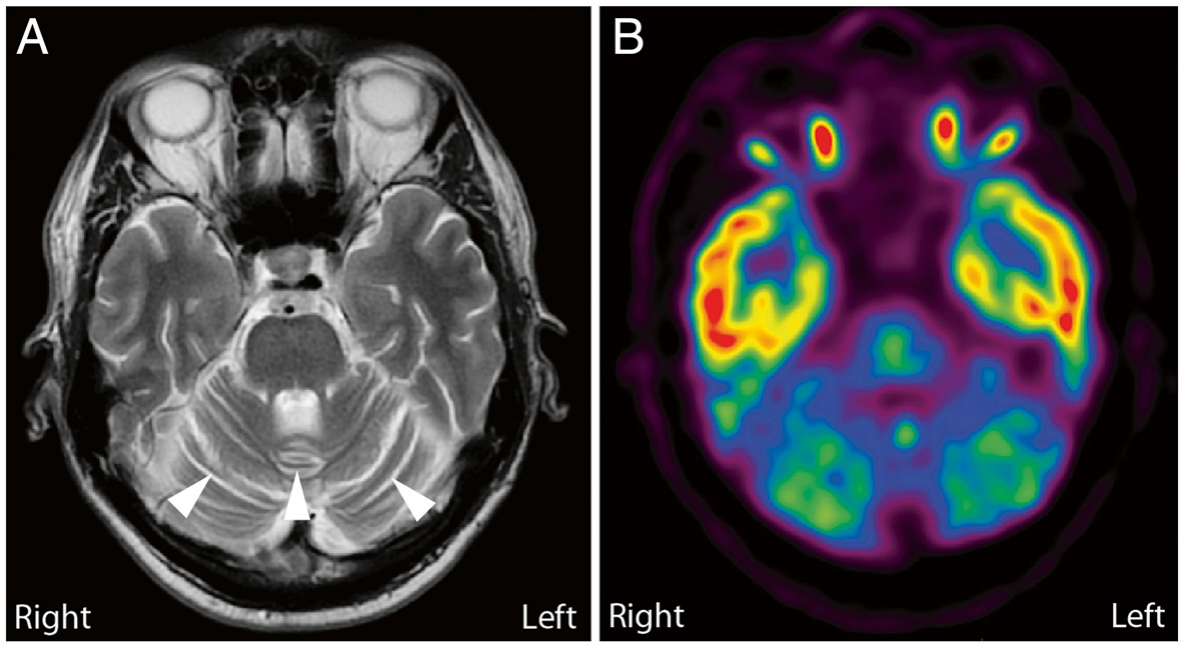
**Brain MRI and**^**18**^ **F-FDG-PET-scan of the proband in the transverse plane. A**: The MRI scan included a diffusion weighted sequence (DWI) and a T2* weighted sequence. No focal lesions were observed; however, the cerebellar hemispheres and vermis appeared atrophic (arrow heads). **B**: ^18^ F-FDG-PET-scan showing strikingly reduced metabolism in the cerebellum suggesting pathology primarily localized to the cerebellum.

No other family member was reported to have symptoms like the proband (Figure 1, III-5); however, the mother (Figure 1, II-4) was diagnosed with multiple sclerosis since the age of 40 with motor deficits and difficulties in coordination as the first signs of disease and only later developed cognitive impairment. She is now severely disabled, wheel chair bound and fully dependent on daily caregivers at age 74. The mother’s brother, living abroad, (Figure 1, II-2) was diagnosed with Parkinson’s disease and the maternal grandfather (Figure 1, I-2) was reported to have died from Parkinson’s disease (Figure 1).

### Clinical features

The proband (Figure 1, III-5) was referred to the hospital at the age of 44. He had worked as a blacksmith until two years before referral, when he was fired due to reduced speed of working. At that time there were no symptoms of cognitive impairment, neither by self reporting nor was it reported by family members. However, over the following 1-1½ years his personality changed and, according to his wife, cognitive impairment evolved rapidly. Furthermore, he also had mild deterioration of motor capabilities and coordination of movements that accelerated 6 months prior to referral. Upon initial clinical examination he was disorientated in time and place. He denied any symptoms, was easily distractible and presented head turning sign and severe word finding difficulties. MMSE score was 14/30. He had horizontal nystagmus, slightly interrupted saccades though with normal initiation, amplitude and velocity, and smooth pursuit of eye movements was impaired. Examination also revealed a fine postural tremor of his hands, a finger tapping test with reduced speed and hand dysdiadokokinesia. He had discrete lower limb ataxia along with a mildly broad based gait, but no upper limb ataxia. Tone was slightly increased in the right leg. Tendon reflexes were brisk in the lower extremities, although the ankle reflexes were absent, and plantar reflexes were flexor. There were no involuntary movements or muscular weakness.

### Biochemistry and Electrophysiology

Blood biochemistry was normal and cerebrospinal fluid protein concentrations and cell counts were normal. Aβ1-42, total-tau, phosphorylated tau-protein, p14-3-3, and neuron specific enolase concentrations were within the normal range and oligoclonal bands were absent.

Standard EEG was abnormal with 3–6 Hz activity over the prefrontal and zygomatico temporal regions observed few times in 2–3 s periodic patterns, but without focal or paroxysmal changes.

### Neuropsychology

As indicated by the MMSE score of 14/30, neuropsychological testing revealed moderate to severe impairment of all cognitive domains and formal testing was difficult. The proband’s attention was drifting and he had problems understanding questions and instructions. He occasionally showed awareness of his difficulties, but did not appear emotionally affected. In contrast, he was smiling almost constantly. Testing showed executive functions to be severely impaired and hardly any test within this domain could be conducted. Episodic memory was affected to a degree where the proband was disorientated in time. However, he was not severely amnesic as he recognized 10/12 pictures among 30 (expected: 12/12) and he was also able to recall episodes which had taken place a few days prior. Language functions were impaired with anomia (inability to name objects), alexia and agraphia. There were signs of acalculia, apraxia and visuospatial impairment (inability to copy simple drawings or assemble simple block designs). Altogether the test profile suggested bilateral cortical involvement with both anterior and posterior cortical areas being severely affected.

### Imaging

I^123^-FP-CIT DAT SPECT was normal (not shown). Brain MRI showed no focal lesions, but the cerebellar hemispheres and vermis appeared atrophic (Figure 2A). The MRI scan included a diffusion weighted sequence (DWI) and a T2* weighted sequence. There was no sign of restricted diffusion as may be seen in Creutzfeldt-Jakob disease or excessive iron accumulation in the basal ganglia which may be suggestive of other causes of neurodegeneraton. ^18^ F-FDG-PET-scan showed strikingly reduced metabolism in the cerebellum (Figure 2B) supporting the cerebellar atrophy visualized by MRI, suggesting pathology localized to the cerebellum. Furthermore, slightly decreased metabolism in the left caudate nucleus was observed, although this finding could only be detected by comparison to scans of normal control individuals with subsequent statistical analyses using NeuroQ software (Philips Healthcare).

### Mutation analyses

The proband’s DNA was screened for mutations in the genes known to cause SCA1, SCA2, SCA3 and SCA6. Due to the phenotypic overlap with many of the polyglutamine diseases analyses for HD and DRPLA were also performed. All were normal. The length of the CAG/CAA repeat in the gene causing SCA17, *TBP*, was determined by fragment analysis of a PCR product amplified from patient DNA using primers 5’-ATG CCT TAT GGC ACT GGA CTG-3’ and 5’-CTG CTG GGA CGT TGA CTG CTG-3’. This revealed a modest expansion of the CAG/CAA repeat of one allele and a CAG/CAA repeat within the normal range on the other allele (43 and 38 CAG/CAA repeats, respectively). After cloning of the PCR products into the PCR2.1 vector (Invitrogen) direct sequencing confirmed the lengths to be 43 and 38. Furthermore, the following repeat structures were observed:

“43”: (CAG)_3_(CAA)_3_(CAG)_11_ CAACAGCAA(CAG)_21_CAACAG

“38”: (CAG)_3_(CAA)_3_(CAG)_8_ CAACAGCAA(CAG)_19_CAACAG

From this it is evident that the patient suffers from SCA17 with repeat structures similar to what has previously been described [4,8,9]. Subsequently, DNA from the patient’s parents were analyzed and showed that the patient’s father (Figure 1, II-5) had two normal alleles of 37 and 38 CAG/CAA repeats, whereas his mother (Figure 1, II-4) had alleles of 37 and 43 CAG/CAA repeats.

## Discussion

Here we report on a patient who was referred to the hospital due to rapid cognitive decline. Due to the severe cognitive impairment and rapid progression, the patient was initially suspected of Creutzfeldt-Jakob disease or a paraneoplastic syndrome. Cerebellar symptoms were not predominant in the clinical picture, but the MRI scan revealed mild atrophy of the cerebellar hemispheres and vermis, and reduced metabolism in the cerebellum visualized by a FDG-PET scan supported the suspicion that the diagnosis should be sought within the cerebellar disorders. The family history revealed a high prevalence of apparently different neurodegenerative disorders based on clinical diagnoses. Considering the phenotypic overlap of many neurodegenerative disorders, this family history was suggestive of a dominantly inherited disorder. Molecular genetic analyses revealed that the proband suffered from SCA17 and that he inherited the disease causing mutation through the maternal line. Diverse phenotypes resembling both HD, Alzheimer’s disease and Parkinson’s disease have previously been reported in patients with short expansions in the *TBP* gene [10,11]. Hence, it is highly likely that the mother’s and the mother’s brother’s diagnoses of multiple sclerosis and Parkinson’s disease, respectively, are incorrect, and that the neurological disorders described in this family represent an example of the broad phenotypic variation of SCA17. Unfortunately, only the proband was available for clinical examination, and the family disease history is based solely on information provided by the proband’s wife.

Cloning of the proband’s alleles of the *TBP* gene showed only marginal expansion of the disease causing CAG/CAA repeat with a length of 43. Considering that alleles with repeat lengths of 42–48 have previously been reported to cause disease with reduced penetrance [5] and that repeat lengths in the shorter end of this range usually cause mild disease progression [5,9,12,13], our case is interesting because of the rapid deterioration observed.

Neuropathology has shown both cerebellar and basal ganglia involvement in SCA17 [14,15], and dopaminergic cell loss has been documented by reduced tracer uptake with SPECT-I123 FP-CIT [14] and Fluoro-dopa PET [16]. Such basal ganglia involvement has not been reported to correlate with symptoms of parkinsonism [17], but it has been suggested to be a function of the severity of the disease with normal uptake of I123 FP-CIT and Fluoro-dopa in pre-clinical stages [14]. Furthermore, basal ganglia involvement has been suggested to be associated with putaminal rim hyperintensity signal on T2 MRI in other cases [6,18,19], and voxel based morphometry has indicated an increased involvement of basal ganglia as the disease progresses [20]. The proband exhibited a rapid and severe decline in cognitive function suggesting an advanced stage of disease, and involvement of the basal ganglia would therefore not have been surprising. However, only slightly decreased metabolism in the left caudate nucleus was observed by the ^18^ F-FDG-PET-scan and we did not see any involvement of basal ganglia on MRI or SPECT-I123 FP-CIT, which emphasizes the heterogeneity of phenotypic presentation and pathology.

The current case emphasizes that diagnosing of neurodegenerative disorders remains a considerable challenge due to the phenotypic overlap, and it shows that the clinical manifestations and the disease course can be different from the classic description of the individual disorders. SCA17 is a very rare disorder and the phenotypic variation is therefore not fully described. Our case contributes to a more detailed characterization of the highly variable clinical picture seen in SCA17, and it highlights the need for a careful clinical and paraclinical examination of patients displaying symptoms of early onset neurodegeneration to enable precise diagnosing on which further counseling and initiation of treatment modalities can be based.

## Conclusion

Here we report on a family with an only marginally expanded CAG/CAA repeat in the *TBP* gene in which the proband presents with severe and rapidly progressing cognitive decline and only mild cerebellar symptoms. The set of symptoms should be considered when diagnosing patients with rapidly progressing cognitive decline.

## Consent

Written informed consent was obtained from the proband and all living relatives for publication of this case report and any accompanying images. A copy of the written consents is available for review by the Editor-in-Chief of this journal.

## Competing interests

The authors declare that they have no competing interests.

## Authors’ contribution

TTN performed the molecular genetic analyses. SM, AL, and JEN performed the clinical investigations of the patient. JEN performed the genetic counseling and obtained blood samples from the family for molecular analyses. JS and SE performed neuropsychological testing. LF and JKN performed the FDG-PET, SPECT-DAT and MRI imaging. TTN, JS, SE, AL, SB, LF, JKN and JEN wrote the paper. All authors approved the paper.

## Pre-publication history

The pre-publication history for this paper can be accessed here:

http://www.biomedcentral.com/1471-2377/12/73/prepub
